# Design and Study of a Novel Thermal-Resistant and Shear-Stable Amphoteric Polyacrylamide in High-Salinity Solution

**DOI:** 10.3390/polym9070296

**Published:** 2017-07-21

**Authors:** Caili Dai, Zhongliang Xu, Yining Wu, Chenwei Zou, Xuepeng Wu, Tao Wang, Xu Guo, Mingwei Zhao

**Affiliations:** School of Petroleum Engineering, State Key Laboratory of Heavy Oil Processing, China University of Petroleum (East China), Qingdao 266580, China; X18353240389@163.com (Z.X.); wuyining@126.com (Y.W.); zoucwupc@163.com (C.Z.); wuxuepeng@163.com (X.W.); wangtao_oilchem@163.com (T.W.); guoxu5597@163.com (X.G.)

**Keywords:** amphoteric polyacrylamide, high-salinity solution, thermal-resistance, shear-stability

## Abstract

Water-soluble polymers are widely used in oilfields. The rheological behaviors of these polymers in high-salinity solution are very important for stimulation of high-salinity reservoirs. In this work, a novel thermal-resistant and shear-stable amphoteric polyacrylamide (PASD), prepared from acrylamide (AM), sodium styrene sulfonate (SSS), and acryloxyethyl trimethylammonium chloride (DAC) monomers, was prepared by free-radical polymerization in high-salinity solution. The amphoteric polyacrylamide was characterized by Fourier transform infrared (FTIR) spectroscopy, nuclear magnetic resonance spectroscopy (^1^H NMR), elemental analysis, thermogravimetric analysis (TG), and scanning electron microscopy (SEM). The amphoteric polyacrylamide exhibited excellent salinity tolerance. The slow increase in apparent viscosity of the polymer with increase in salinity was interesting. The amphoteric polyacrylamide showed perfect temperature resistance in high-salinity solution. The viscosity retention reached 38.9% at 120 °C and was restored to 87.8% of its initial viscosity when temperature was decreased to room temperature. The retention ratio of apparent viscosity reached 49.7% at 170 s^−1^ and could still retain it at 25.8% at 1000 s^−1^. All these results demonstrated that PASD had excellent thermal-resistance and shear-stability in high-salinity solution. We expect that this work could provide a new strategy to design polymers with excellent salinity-tolerance, thermal-resistance, and shear-stability performances.

## 1. Introduction

Water-soluble polymers have been intensively explored, due to their practical importance as viscosity-enhancing agents, flocculating agents, food additives, etc. [1,2]. Development of new types of water-soluble polymers is an important subject in current research. Polyacrylamide has been successfully employed in wastewater treatment, the papermaking industry, and the oil industry due to its thickening ability, flocculation and rheological behaviors [3,4]. However, the properties of the polymeric solution render it sensitive to changes in the external environment, such as temperature and salinity [5]. Improving the resistance of polyacrylamide to its external environment is an effective way to broaden its applications in harsh environmental conditions.

Water-soluble polymers have been successfully employed in oilfields. To date, polyacrylamide (PAM) and partially hydrolyzed polyacrylamide (HPAM) have been the most widely used polymers in oil fields, such as in chemical flooding, drilling fluids, fracturing fluids, clay stabilizers, and other EOR technologies [6,7,8,9]. However, there are some limitations associated with the application of water-soluble polymers in reservoirs under harsh conditions. PAM and HPAM show serious losses in their apparent viscosities, due to the hydrolysis of –CONH_2_ groups above 70 °C [10]. Multivalent metal ions react with the –COO^−^ groups, leading to flocculation in high-salinity solutions [11,12]. High shear rates can destroy the linear chain structures of PAM and HPAM [13]. PAM and HPAM are not suitable for stimulation of high temperature or high-salinity reservoirs. Pioneering work indicated that acrylamide (AM) copolymerized with suitable ionic monomers could significantly improve its performance [14,15,16]. A large number of acrylamide-based copolymers synthesized for EOR were based on this strategy [17]. Various ionic monomers were copolymerized with AM, in order to modify the properties of the copolymers [18,19], which included anionic as well as cationic monomers. The anionic monomers included sodium-2-acrylamido-2-methylpropane sulfonate (NaAMPS), sodium acrylate (NaAA), and sodium-3-acrylamido-3-methylbutanoate (NaAMB) [20,21,22]. The cationic monomers included 2-(acrylamido)-2-methylpropanetrimethylammonium chloride (AMPTAC), diallyl dimethyl ammonium chloride (DMDAAC), dimethyldodecyl (2-acrylamidoethyl) ammonium bromide (DAMAB), and 2-acrylamido-2-methylpropanedimethylammonium chloride (AMPDAC) [23,24,25]. The cationic or anionic polyacrylamide exhibited perfect temperature resistance and thickening ability in fresh water. Copolymers bearing only positive or negative charges possess only electrostatic repulsive forces in their molecular chains. The polymer chain expands to have a large hydrodynamic volume in fresh water. These copolymers have the highest viscosity in fresh water. However, when a cationic or an anionic acrylamide copolymer is dissolved in a saline solution, there is an obvious reduction in its viscosity. The presence of salts weakens the intramolecular repulsive interactions, the molecular chains curl, and the hydrodynamic volume shrinks. The viscosity of the traditional acrylamide copolymer reduces sharply with an increase in salinity. Still the sensitivity to salinity limits the applications of these polymers in high-salinity reservoirs.

Recently, polyampholyte has attracted a lot of attention due to its unique properties [26]. A polyampholyte contains both positive and negative charges attached to the same polymeric backbone. The viscosity of a polyampholytic solution increases with increase in salinity under certain conditions, popularly known as anti-polyelectrolyte behavior in solution [27]. Polyampholytes have a wide range of applications such as ion exchange and chelation materials to bind trace metals from drinking water, sewage treatment, paper reinforcement, soil conditioning, pigment retention, and in formulations of shampoos and hair conditioners [28,29]. Polyampholytes have also been used in petroleum explorations. For instance, He [30] synthesized an amphiphilic copolymer containing acrylamide, acrylic acid, 2-acrylamido-2-methyl propane sulfonic acid, and modified β-cyclodextrin to be an oil displacement agent, which showed good temperature resistance and oil displacement ability. Patrizio Raffa [31] also reported amphiphilic copolymers based on poly(ethylene glycol) methyl ether acrylate (PEGA) as the polymeric surfactant for applications in enhanced oil recovery. Favero [32] synthesized a high molecular-weight polyampholyte containing acrylamide, sodium acrylate, and diallyl dimethyl ammonium chloride as a fracturing fluid thickener. These studies indicate that the polyampholyte has excellent thickening ability in solution at high temperatures. However, effective thickening of a high-salinity solution is still a big challenge.

This paper presents the design and synthesis of an amphoteric polyacrylamide. This polyampholyte was synthesized by polymerization in an aqueous solution. The characteristics of the functional group decide the properties of the polymer to a great extent [33]. However, in addition to this, the reactivities of the comonomers should also be considered. The anionic monomer, sodium styrene sulfonate (SSS), possesses temperature resistant headgroups, which include both, the –SO_3_^−^ group and the aromatic ring. Acrylamide copolymer containing SSS is expected to exhibit remarkable anti-shearing property and temperature resistance [22]. The cationic monomer, acryloxyethyl trimethyl ammonium chloride (DAC), can inhibit the hydration swelling ability of clay minerals, and its bulky side chains can boost the salinity tolerance and shear-stability [34]. Furthermore, both SSS and DAC are cheap and undergo copolymerization actively. Hence, by employing the above design strategy, the amphoteric polyacrylamide (PASD) was copolymerized from AM, SSS, and DAC under optimized reaction conditions. A series of experiments were carried out to investigate the structure and characteristics of the copolymer. The detailed rheological properties in high-salinity solution have also been studied.

## 2. Materials and Methods

### 2.1. Materials

AM (99.0%, AR) was purchased from Aladdin Industrial Co. Ltd. (Shanghai, China). SSS (99.0%, AR) was purchased from Energy Chemical Co. Ltd. (Shanghai, China). DAC with a purity of 80 wt % in water was purchased from J&K Scientific Co. Ltd. (Beijing, China). Ammonium persulfate (APS), sodium bisulfite (NaHSO_3_), sodium chloride (NaCl), calcium chloride (CaCl_2_), magnesium chloride (MgCl_2_), and ethanol (C_2_H_5_OH) were all of analytical reagent grade and were obtained from Sinopharm Chemical Reagent Co. Ltd. (Shanghai, China). The chemicals were used directly without further purification. Deionized water was used throughout this work.

### 2.2. Synthesis of Polyampholyte PASD

**Scheme 1:**
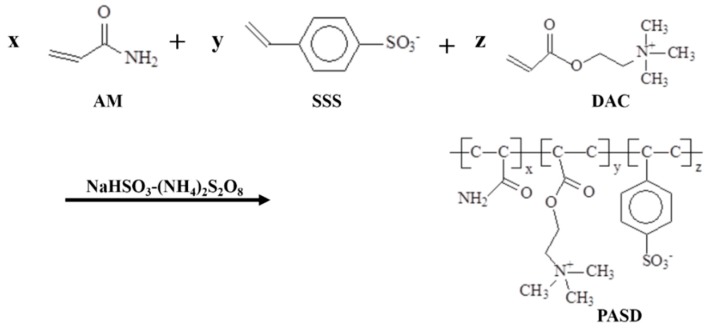
Synthesis of amphoteric polyacrylamide PASD.

AM (20.39 g) and SSS (12.85 g) were dissolved in deionized water (102 mL). DAC (14.87 g) was added to the above solution under inert nitrogen atmosphere. NaOH solution (10 wt %) was added to the above solution to adjust the pH to 6. The reaction mixture was heated to 35 °C and stirred for 30 min. Then, the initiators APS (0.095 g) and NaHSO_3_ (0.040 g) in molar ratio of [n(APS)/n(NaHSO_3_) = 1.1/1] were added drop-wise. The copolymerization was carried out at 35 °C under nitrogen atmosphere for 4 h. After cooling to room temperature, it was washed several times with 4:1 (*v*/*v*) mixture of ethanol and water, and dried at 60 °C under vacuum. Thereafter, the sample was screened, and finally the copolymer product in powdered form was obtained. The synthesis of PASD is shown in Scheme 1.

### 2.3. Conversion Measurement

Conversion of the monomers to the copolymer was calculated from the masses of the copolymer and monomers, determined gravimetrically. Samples were withdrawn from the reaction mixture periodically and washed several times with a 4:1 (*v*/*v*) mixture of ethanol and water to remove the unreacted monomers. Then, samples were dried and then weighed. The conversion of the monomers to the copolymer (*C* %) was calculated using Equation (1):

(1) $$C\;\%=\left(\frac{W_{1}}{W_{2}}\right)\times \left(\frac{1}{W}\right)\times 100\%$$

where, *W*_1_ and *W*_2_ are the weights of dry polymer, reaction mixture, and *W* is the weight percent of total monomer in initial reaction mixture.

### 2.4. Characterizations

Fourier transform infrared (FTIR) spectrum of the dried and powdered PASD was recorded on a Nicolet 6700 FTIR spectrometer (Nicolet, WI, USA) following the KBr-disk method. The sample was mixed with KBr powder, pressed into a disk, and then dried at 105 °C for 24 h prior to the analysis. The spectrum was recorded at room temperature in the range of 4000–500 cm^−1^, with minimum 32 scans at a resolution of 4 cm^−1^. Nuclear magnetic resonance spectrum (^1^H NMR) of PASD (in D_2_O) was obtained on a Bruker AV III-400 NMR spectrometer (Bruker Biospin, Switzerland) that operated at 400 MHz. Elemental analysis was performed using Vario EL-III elemental analyzer (Elementary Analyze System GmbH, Hanau, Germany). Thermogravimetric analysis (TG) of PASD was conducted on the STA 449 F3 Jupiter instrument (Netzsch, Bavaria, Germany). The sample was heated at a rate of 10.0 °C/min from 30 to 600 °C under nitrogen atmosphere. The surface morphology of PASD in a high-salinity solution was investigated by scanning electron microscopy (SEM, S-4800, Hitachi, Tokyo, Japan). The sample contained 2% PASD in 150,000 mg/L NaCl solution. The solution was dropped onto a special glass and rapidly frozen in liquid nitrogen. The surface of the sample was observed using a scanning electron microscope that operated at an accelerating voltage of 5 kV.

### 2.5. Properties of the PASD

#### 2.5.1. Salinity-Tolerance Test

The salinity tolerance of PASD solution was tested using NaCl, CaCl_2_, and MgCl_2_. The apparent viscosities of polyampholyte solutions in different saline concentrations were determined by HAAKE MARS 60 rotational rheometer (Thermo, Karlsruhe, Germany). The apparent viscosities were determined using the cone/plate geometry (diameter 35 mm, angle 1°, plate to plate gap 0.052 mm) under a shear rate of 7.34 s^−1^ at room temperature.

#### 2.5.2. Temperature Resistance Test

The viscosities of 2.5% PASD in 150,000 mg/L NaCl solution at both room temperature and high temperatures were determined using HAAKE MARS 60 rotational rheometer. The steady shear viscosities were obtained at a heating rate of 3 °C/min in the temperature range from 25 to 120 °C, under a shear rate of 170 s^−1^, using the high pressure test system (D300 cell, PZ38 rator).

#### 2.5.3. Shear Stability Test

The shear resistance tests were performed at different shear rates and the shear stress was measured from 0.1 to 1000 s^−1^. The sample containing 3% PASD in 150,000 mg/L NaCl solution was tested using HAAKE MARS 60 rotational rheometer, with coaxial cylinder geometries (CC41/Ti Rotor) at 25 °C.

## 3. Results and Discussion

### 3.1. Effects of Reaction Conditions

**Figure 1:**
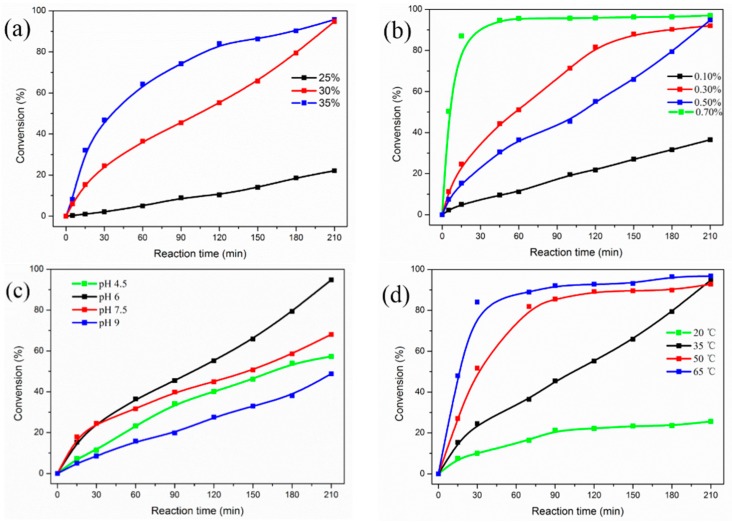
Conversion versus time curves showing the effects of reaction conditions on conversion. Effect of: (**a**) total monomer concentration, (**b**) initiator concentration, (**c**) pH, and (**d**) reaction temperature.

The effects of reaction conditions on the conversion of the monomers to PASD were investigated by the single factor method. The curves for conversion versus time under different reaction conditions are shown in Figure 1. From Figure 1a, it is evident that the polymerization rate increased with increase in monomer concentration. The more number of active molecules present in a solution containing higher monomer concentration increased the probability of reaction. There was a dramatic increase in temperature and viscosity, which indicated that gelation increased with increase in monomer concentration.

Initiator plays a key role in polymerization. Figure 1b shows conversion as a function of time at different initiator concentrations. The polymerization rate increased constantly as the concentration of the initiator increased. The number of free radicals and active aggregates increased with increase in initiator concentration. An initiator concentration of 0.3 wt % was found to be optimum.

Figure 1c shows the influence of pH on the conversion of monomers to PASD. It was clear from the figure that a lower or higher pH value decreased the polymerization rate. The maximum polymerization rate and final conversion were achieved when the pH was 6.

The effect of temperature on the conversion of monomers to PASD can be seen in Figure 1d. The polymerization rate increased with increase in temperature. This could be attributed to the rapid increase in the decomposition rate of the initiator as well as an increase in the propagation rate constant. The polymerization occurred explosively at a very high temperature. However, at a very low temperature, the yield and molecular weight of the copolymer were not satisfactory. The optimum temperature for the polymerization reaction was found to be 35 °C.

A higher final conversion and a stable reaction rate are very important for the preparation of the polymer. Finally, the optimal reaction conditions were: a total monomer concentration of 30 wt %, initiator concentration of 0.3 wt %, pH of 6, and a reaction temperature of 35 °C. A near-linear relationship between the conversion-time curves and the highest final conversion could be reached under these reaction conditions. The near-linear relationship of the conversion-time curves is beneficial for industrial production.

### 3.2. Characterization of Copolymers

**Figure 2:**
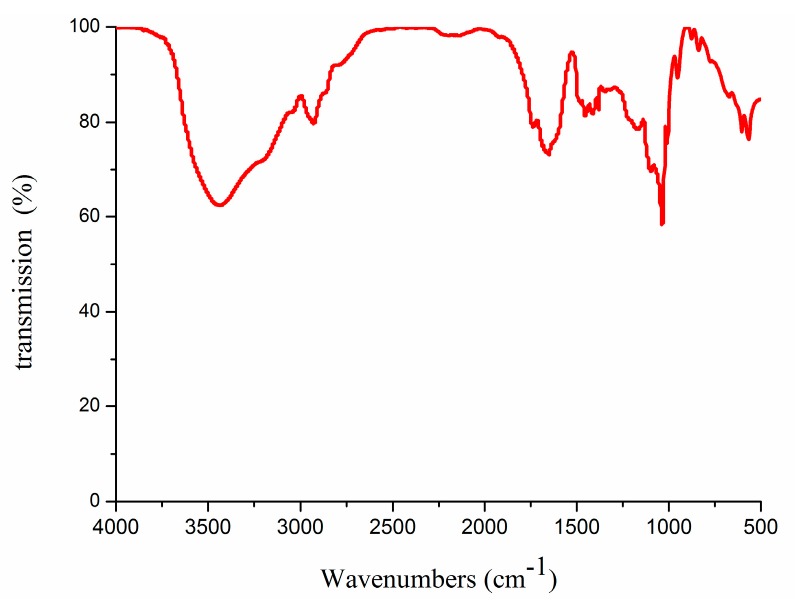
FTIR spectrum of PASD.

The FTIR spectrum of the synthesized PASD copolymer is shown in Figure 2. The peak at 1685 cm^−1^ could be assigned to the C=O stretching vibrations of the –CONH_2_. The broad intense peak at 3435 cm^−1^ was due to the N–H stretching vibrations of the –CONH_2_ group. These characteristic peaks confirmed the successful introduction of AM into the copolymer. Characteristic peaks at 2930 and 2871 cm^−1^ were attributed to the stretching vibrations of –CH_3_. The peaks at 2850 and 1410 cm^−1^ were assigned to the –CH_2_– groups on the polymeric chain, whereas the stretching vibrations of the ester –COO^−^ were observed at 1735 cm^−1^. The presence of these peaks indicated that DAC was successfully incorporated into PASD. Peaks at 1036 and 1170 cm^−1^ were characteristic of the –SO_3_^−^ group. The peak at 620 cm^−1^ also proved the presence of –SO_3_^−^ group. Characteristic peaks of C=C of benzene ring appeared at 1493 and 1454 cm^−1^. Peaks at 860 and and 820 cm^−1^ were characteristic of para-substituted aromatic rings. The appearance of these peaks suggested the successful copolymerization of DAC. The FTIR spectrum of the copolymer confirmed that the polyampholyte PASD was synthesized successfully.

**Figure 3:**
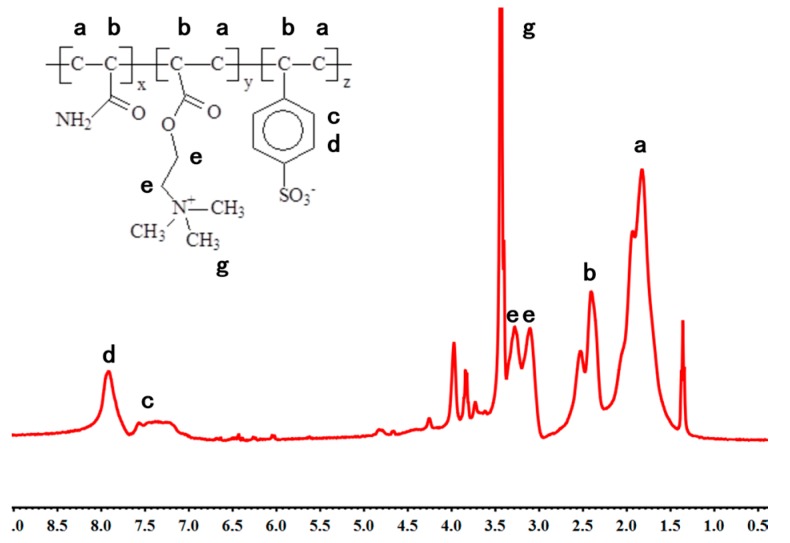
^1^H NMR spectrum of PASD.

^1^H NMR analysis further verified the successful preparation of PASD. The ^1^H NMR spectrum of PASD is shown in Figure 3. The peak at 7.91 ppm was due to the protons of the aromatic ring, indicating the presence of SSS. Peaks at 3.28 and 3.11 ppm were assigned to the –CH_2_– groups of DAC attached to –N(CH_3_)_3_^+^, whereas the characteristic peak of methyl –CH_3_ groups of DAC appeared at 3.44 ppm.

Elemental analysis was conducted to evaluate the components of PASD. At high temperature, in the presence of oxygen and catalyst, S and N elements of the sample were oxidized to SO_2_ and N_2_, respectively. The gases were separated in the chromatographic column by the carrier gas. Finally, the different elements were detected in the thermal conductivity cell and analyzed. Based on the principles of mass conservation and the element conservation, the ratios of different monomers in the copolymer were calculated. The results are shown in Table 1. The composition of the resultant copolymer was based on several factors, which included monomer concentrations and their corresponding reactivity ratios. It was observed that PASD contained an almost equivalent number of cationic and anionic monomers. The properties and rheological behavior of PASD in a high-salinity solution are mainly discussed in the subsequent sections.

**Figure 4:**
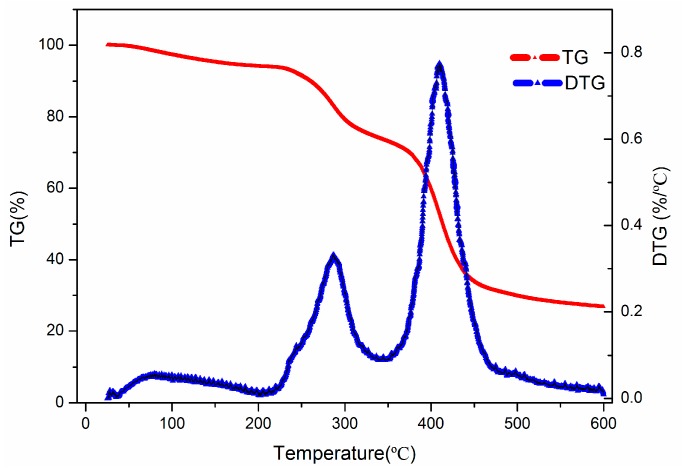
TG and DTG curves of PASD.

The thermogravimetric (TG) and differential thermogravimetric (DTG) curves of PASD are shown in Figure 4. In general, PASD experienced three stages of weight loss in both TG and DTG. A weight loss of 5.49% in the first stage in the temperature range of 30–198 °C could be attributed to the removal of intermolecular or intramolecular moisture. The second stage, with a weight loss of 11.94%, occurred in the temperature range of 198–326 °C, due to the decomposition of amide groups. The third stage accounted for a weight loss of 48.66% in the temperature range of 326–500 °C, as a result of the decomposition of the quaternary ammonium salt and benzenesulfonamide groups. The residue of PASD at 600 °C was 30%. Compared to the TG curve of HPAM reported earlier [35], the decomposition temperature of PASD was found to be higher. The aromatic ring made PASD extraordinary rigid and stable. The –SO_3_^−^ group was also stable at high temperature. The presence of aromatic rings and –SO_3_^−^ groups significantly improved the thermal stability of PASD. 

**Figure 5:**
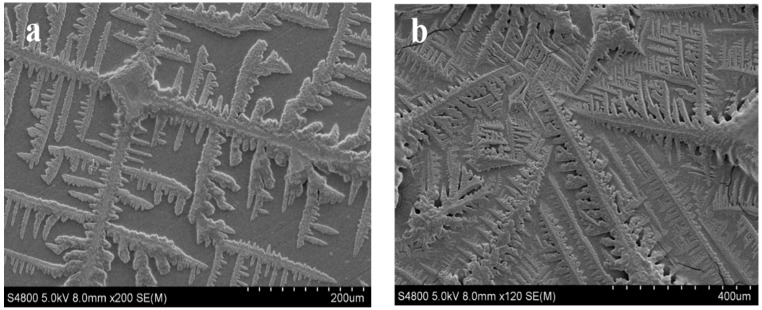
Scanning electron microscopic (SEM) images of PASD in salinity solution: (**a**) sample solution at 200 μm, (**b**) sample solution at 400 μm.

The SEM images of 2% copolymer in a high-salinity (150,000 mg/L) NaCl solution are shown in Figure 5. The microstructure of PASD polymer can be seen clearly in Figure 5a. The amphoteric polyacrylamide showed a fish-bone framework structure, with a completely expanded polymeric coil in the high-salinity solution. The compact microscopic structures can be seen in Figure 5b. A large number of intermolecular linkages could be seen between the polymer chains and the high hydrodynamic volume was a result of these dense intermolecular linkages. The perfect thickening ability of the amphoteric polyacrylamide could be mainly attributed to the expansion of the polymeric coil and the large number of intermolecular linkages in the high-salinity solution. The anti-polyelectrolyte behavior of PASD was strongly demonstrated by SEM.

### 3.3. Salinity Tolerance Ability

**Figure 6:**
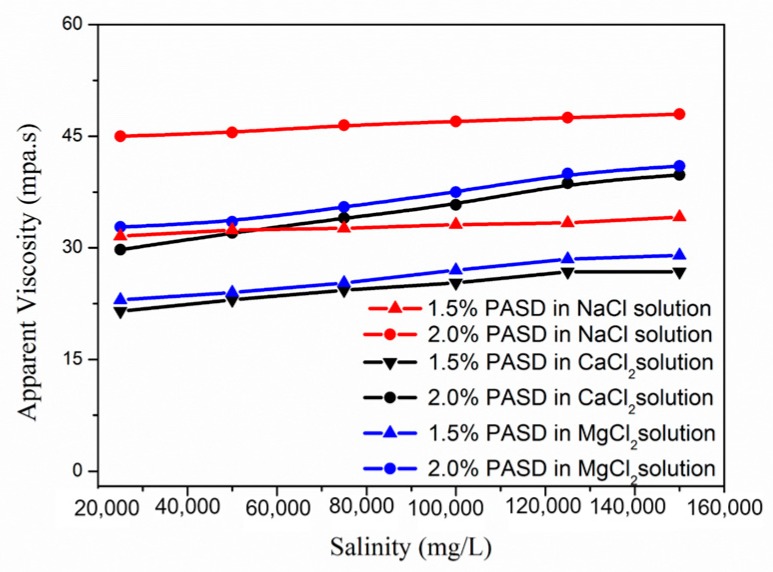
Effect of salinity concentration on apparent viscosity of PASD solution.

The apparent viscosities of PASD solutions at different salinities are shown in Figure 6. The polymer concentrations were 1.5% and 2.0%. The viscosity of PASD solution increased slowly with increase in salinity. This was contrary to the traditional AM copolymer, where the viscosity reduced drastically with increase in salinity. Compared to the traditional AM copolymer, PASD showed excellent anti-polyelectrolyte behavior in high-salinity solutions.

**Figure 7:**
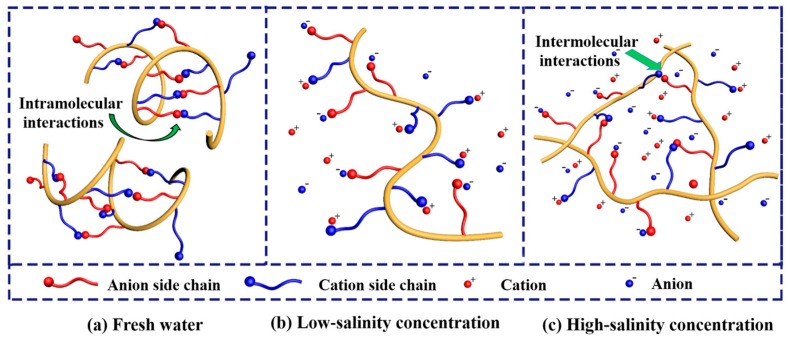
Thickening mechanism of PASD chains in (**a**) fresh water; (**b**) the low-salinity solution; and (**c**) the high-salinity solution.

The mechanism of thickening is shown in Figure 7. The amphoteric polyacrylamide exhibited strong intramolecular electrostatic interactions, due to the co-existence of positive and negative charges. The intramolecular electrostatic interactions dominated the polyampholytic properties and the charges prevented the expansion of the polymeric coil in fresh water. The hydrodynamic volume reduced drastically, which lowered the apparent viscosity of PASD solution. The inorganic ions had stronger electrostatic interactions with the ionic monomer. With increase in salinity, the intramolecular interactions reduced. The hydrodynamic volume of PASD increased, and the apparent viscosity increased slightly. With further increase in salinity, the intermolecular interactions dominated the polyampholytic properties. The intermolecular interactions strongly increased the apparent molecular weight of the system, as depicted by SEM image in Figure 5b. Thus, the apparent viscosity of PASD continued to increase in high-salinity solutions. It was observed that the overall viscosity of PASD was lower than that of the acrylamide copolymer reported previously [35]. This could be simply due to the lower molecular weight of PASD. It was more likely that the coil was greatly shrunk due to the large number of intramolecular crosslinks, resulting in an apparent lowering of molecular weight. It should be noted that the hydrodynamic volume would continue to increase even in media of higher ionic strength. This excellent salinity tolerance of PASD can be very useful in production of high-salinity reservoirs.

### 3.4. Temperature Resistance Ability

**Figure 8:**
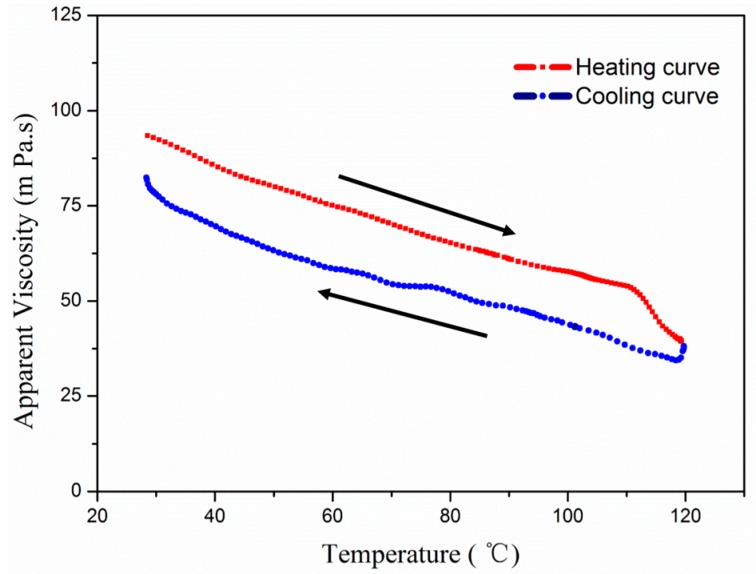
Effect of temperature on the apparent viscosity of PASD in NaCl solution.

The effect of temperature on the apparent viscosity of PASD in NaCl solution is shown in Figure 8. The apparent viscosity reduced gradually with increase in temperature. The retention rate of the copolymer solution reached 38.9% at 120 °C, with a viscosity of 37 mPa·s. The –SO_3_^−^ headgroup displayed stronger hydrogen bonding than the –COO^−^ group, resulting in remarkable thermal stability at high temperature. The copolymer solution showed outstanding thermal stability and thickening ability at high temperature, due to the presence of benzene rings and sulfonate groups. With decrease in temperature, the apparent viscosity began to increase, and the viscosity reached an 87.8% retention rate, compared to its initial viscosity at 25 °C. PASD had excellent temperature resistance in high-salinity solution.

### 3.5. Shear Stability

**Figure 9:**
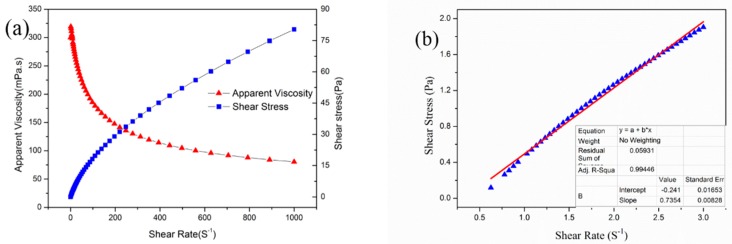
Shear resistance of PASD in NaCl solution: (**a**) effect of shear rate on apparent viscosity of sample; (**b**) effect of shear rate on shear stress in log–log scale.

The shear stability of PASD in NaCl solution was investigated, as shown in Figure 9. The relation between shear rate and shear stress was calculated using Equation (2). The consistency coefficient (κ) and power law exponent (*n*) were obtained from Figure 9b, which showed the relationship between shear rate and shear stress on the log scale. The *n* and κ values of amphoteric polyacrylamide were 0.74 and 0.57 Pa·s^0.74^, respectively. The PASD solution exhibited an obvious pseudoplastic fluid behavior.

(2) $$\tau ={\kappa \gamma }^{n}$$

where τ was shear stress, Pa; γ was shear rate, s^−1^; κ was the consistency coefficient, Pa·s^n^; and *n* was power law exponent.

It became clear that the apparent viscosity of polyampholyte solution decreased sharply at a shear rate of 3.7–200 s^−1^. However, there was a slight change in the apparent viscosity at a higher shear rate of 200–1000 s^−1^. PASD showed a viscosity retention rate of 25.8% at 1000 s^−1^. The intermolecular linkages played a key role in the thickening ability of PASD in a high-salinity solution. A high shear rate had less impact on the on the breaking of the intermolecular linkages in the polymer. Hence, PASD displayed extraordinary shear stability in a high-salinity solution and high viscosity retention at 1000 s^−1^.

## 4. Conclusions

In this study, a novel amphoteric polyacrylamide, PASD, was successfully synthesized through free-radical polymerization in aqueous solution. The effects of reaction conditions on the reaction rate and final conversion were investigated using the single factor method. The optimal reaction conditions were established at mild conditions: a total monomer concentration of 30 wt %, initiator concentration of 0.3 wt %, pH of 6, and a reaction temperature of 35 °C. FTIR and ^1^H-NMR spectroscopic analyses proved qualitatively that the structure of PASD included AM, DAC, and SSS moieties. Elemental analysis was used to quantitatively determine the composition of PASD. The mole ratios of the monomers, AM:SSS:DAC, in the copolymer were 64.9%:17.6%:17.5%. This implied that PASD contained almost an equivalent number of cationic and anionic monomers. The TG characterization was used to test the thermal stability of PASD, it showed that the PASD had a higher decomposition temperature compared to traditional polyacrylamide due to the presence of an aromatic ring and –SO_3_^−^ groups. From the results of salinity tolerance test and element analysis, besides the SEM images of PASD in high-salinity solution, the thickening mechanism of PASD in high-salinity solutions was proposed. This unique polyampholyte structure also showed enhanced temperature resistance and shear stability in high-salinity solution. The viscosity retention reached 38.9% at 120 °C. The retention ratio of apparent viscosity reached 49.7% at 170 s^−1^ and could still retain it at 25.8% at 1000 s^−1^. PASD has excellent thickening ability in high-salinity solution. It may be widely applied in drilling fluid and superabsorbent polymers. PASD also has remarkable salinity tolerance, temperature resistance, and shear stability. It has great advantages for the additives of flocculants, coatings and cosmetics.

## Figures and Tables

**Table 1:** Elemental analysis and composition of PASD.

	Comonomer used			Comonomer ratios in the feed (mole %)			Percentages of elements (wt %)		Comonomer ratios in the copolymer (mole %)		
Sample	A	B	C	A	B	C	S	N	A	B	C
PASD	AM	SSS	DAC	70	15	15	4.84	9.93	64.9	17.6	17.5
